# Outcomes of a Diagnostic Pathway for Prostate Cancer Based on Biparametric MRI and MRI-Targeted Biopsy Only in a Large Teaching Hospital

**DOI:** 10.3390/cancers15194800

**Published:** 2023-09-29

**Authors:** Leonor J. Paulino Pereira, Daan J. Reesink, Peter de Bruin, Giorgio Gandaglia, Erik J. R. J. van der Hoeven, Giancarlo Marra, Anne Prinsen, Pawel Rajwa, Timo Soeterik, Veeru Kasivisvanathan, Lieke Wever, Fabio Zattoni, Harm H. E. van Melick, Roderick C. N. van den Bergh

**Affiliations:** 1 Department of Urology, St Antonius Hospital, 3435CM Nieuwegein, The Netherlandsp.bruin@antoniusziekenhuis.nl (P.d.B.); h.van.melick@antoniusziekenhuis.nl (H.H.E.v.M.); r.van.den.bergh@antoniusziekenhuis.nl (R.C.N.v.d.B.); 2 Unit of Urology, Division of Oncology, Gianfranco Soldera Prostate Cancer Laboratory, IRCCS San Raffaele Scientific Institute, 20132 Milan, Italy; 3 Department of Urology, Città della Salute e della Scienza, University of Turin, 10124 Turin, Italy; 4 Department of Urology, Comprehensive Cancer Center, Medical University of Vienna, 1090 Vienna, Austria; 5 Department of Urology, Medical University of Silesia, 41-800 Zabrze, Poland; 6 Division of Surgery and Interventional Science, University College London, London WC1E 6BT, UK; 7 Urologic Unit, Department of Surgery, Oncology and Gastroenterology, University of Padova, 35122 Padua, Italy

**Keywords:** prostate cancer, MRI, biopsy, Gleason, targeted, systematic

## Abstract

**Simple Summary:**

Prostate cancer (PCa) is diagnosed with tissue biopsy. The biopsy strategy may include needle cores covering the whole gland randomly and/or targeting abnormalities on imaging. This process aims to detect dangerous PCa; small cancers need not to be diagnosed. When deciding on biopsy and the biopsy strategy, an MRI scan has become an increasingly important tool. MRI can be used to decide to biopsy and to potentially use targeted biopsy cores only. A database of men suspected of PCa was used. It found that when solely relying on MRI to biopsy, 34% fewer men would undergo biopsy, but 7% of the potentially dangerous tumors would not have been detected. With targeted biopsy cores only, 75% fewer needle cores would be needed, but another 9% of potentially dangerous PCa would remain undetected. These missed lesions were usually the smaller ones. This information is helpful to balance the pros/cons of an MRI-based pathway.

**Abstract:**

Background: Diagnostic pathways for prostate cancer (PCa) balance detection rates and burden. MRI impacts biopsy indication and strategy. Methods: A prospectively collected cohort database (N = 496) of men referred for elevated PSA and/or abnormal DRE was analyzed. All underwent biparametric MRI (3 Tesla scanner) and ERSPC prostate risk-calculator. Indication for biopsy was PIRADS ≥ 3 or risk-calculator ≥ 20%. Both targeted (cognitive-fusion) and systematic cores were combined. A hypothetical full-MRI-based pathway was retrospectively studied, omitting systematic biopsies in: (1) PIRADS 1–2 but risk-calculator ≥ 20%, (2) PIRADS ≥ 3, receiving targeted biopsy-cores only. Results: Significant PCa (GG ≥ 2) was detected in 120 (24%) men. Omission of systematic cores in cases with PIRADS 1–2 but risk-calculator ≥ 20%, would result in 34% less biopsy indication, not-detecting 7% significant tumors. Omission of systematic cores in PIRADS ≥ 3, only performing targeted biopsies, would result in a decrease of 75% cores per procedure, not detecting 9% significant tumors. Diagnosis of insignificant PCa dropped by 52%. PCa undetected by targeted cores only, were ipsilateral to MRI-index lesions in 67%. Conclusions: A biparametric MRI-guided PCa diagnostic pathway would have missed one out of six cases with significant PCa, but would have considerably reduced the number of biopsy procedures, cores, and insignificant PCa. Further refinement or follow-up may identify initially undetected cases. Center-specific data on the performance of the diagnostic pathway is required.

## 1. Introduction

An optimal diagnostic pathway for patients referred with a suspicion of prostate cancer (PCa) should detect significant tumors while minimising the detection of insignificant lesions, with the least possible patient burden and costs for the healthcare system. MRI of the prostate may impact the diagnostic pathway with a favourable effect on both the indication for prostate biopsy, as well as the biopsy strategy itself [1].

By only performing prostate biopsy in men with an abnormal MRI, roughly 25% fewer patients undergo prostate biopsy when compared to a fixed PSA threshold [2]. However, MRI misses around 10% of ISUP grade group (GG) ≥ 2 tumors [3]. Risk calculators, such as the ERSPC prostate risk-calculator, are another way of improving the indication for prostate biopsies [4]. MRI and risk-calculator may be combined or sequenced, in order to optimize the biopsy risk stratification. However, choices in primary risk stratification tools such as the MRI or risk-calculator, and the sequence in which these are applied, could impact the number of biopsy procedures and insignificant and significant PCa detection rates [5].

European guidelines now recommend performing an MRI after biopsy indication, before proceeding with prostate biopsies [1]. Depending on MRI findings, the biopsy cores can be performed systematically, MRI-lesion targeted, or combined. As presented in a recent review by Connor et al., MRI-targeted biopsies alone detect higher rates of clinically significant PCa (up to 18%) and lower rates of insignificant PCa compared to systematic biopsies [6,7]. Omitting systematic biopsies may miss 10–15% of the significant cancers when compared to the combined approach [8,9]. Combined prostate biopsies yield the highest detection rates of clinically significant PCa [6]. Furthermore, combined prostate biopsies also provide a better estimation of prostatectomy pathology outcomes, although this difference is limited, and is mainly due to GG 2 (Gleason 3 + 4 = 7) tumors [9]. In MRI PIRADS 5 lesions, the added value of systematic biopsies is relatively limited [10].

The Dutch 4M study presented the results of a combined diagnostic pathway, exploring the impact of using prostate MRI as the only indication for prostate biopsy, and only taking targeted cores [11]. The omission of systematic biopsy in negative MRI cases missed 4% of the significant cancers. Targeted biopsies missed 11% of the significant cases, but were considered non-inferior, while the detection rate of insignificant was drastically reduced [11]. However, real-life data is scarce.

Therefore, this study presents the outcomes of applying a hypothetical targeted-only biopsy approach in a large cohort of men referred for the suspicion of PCa, who underwent systematic and targeted biopsy when the risk-calculator score was >20% or the MRI score was PIRADS ≥ 3, using biparametric MRI.

## 2. Patients and Methods

### 2.1. Patients

The study cohort comprised all men referred by the general practitioner (GP) to the St. Antonius Hospital in Utrecht, The Netherlands, between August 2018 and April 2019, for the suspicion of PCa based on an elevated PSA and/or abnormal DRE. Generally, the GP applied a PSA threshold of 3.0 ng/mL. Exclusion criteria for the cohort included previous MRI of the prostate, prostate biopsies, MRI contra-indications, or using 5-alpha reductase inhibitors. Patients with a priori high risk of metastasized disease (defined as PSA > 50 ng/mL) were also excluded. The study was approved by the medical-ethical committee (“MECU”) of St. Antonius Hospital (NL64381.100.17) and completed according to the “good clinical practice” guidelines.

### 2.2. Protocol

All referred patients underwent direct bi-parametric prostate MRI. In addition, the ERPSC risk-calculator was completed to score the risk of a positive prostate biopsy [4]. In line with the protocol, prostate biopsies were performed when at least the risk calculator was ≥20% or the MRI PIRADS score was ≥3. Therefore, patients with risk-calculator < 20% and MRI PIRADS 1–2 did not undergo biopsy. The outcomes of this standard diagnostic pathway were used as a reference. This pathway consists of standard MRI for all referred patients, with a wide indication for biopsy (based on risk-calculator and/or MRI), and with the combination of targeted plus systematic cores when possible. 

### 2.3. Biopsy

After the biopsy indication was made, the protocol advised taking both targeted biopsy cores (minimum 2 cores per MRI lesion) and systematic biopsy cores (8, 10, or 12, depending on prostate volume <40, 40–60, and >60 mL, respectively). Biopsy cores were MRI-TRUS targeted according to cognitive fusion [12]. During the study period, the standard method for prostate biopsy was the transrectal approach. The biopsies were performed by experienced urologists (>100 biopsy procedures) using a “biplane” TRUS-probe (Hitachi Healthcare Americas, Twinsburg, OH, USA). Biopsy cores were analyzed by experienced pathologists, who specialized in uropathology and reviewed according to the ISUP 2014 protocols [13]. Significant PCa was defined as GG 2 or higher (Gleason score ≥ 3 + 4 = 7) in at least one prostate biopsy core.

### 2.4. Risk-Calculator

The risk for positive prostate biopsy was calculated using the ERPSC risk-calculator number 3 [14]. This calculator incorporates PSA, DRE findings, prostate volume, and TRUS lesions to predict the detection of PCa and high-grade PCa at (systematic) biopsy. MRI calculated prostate volume was used. MRI PIRADS results were not incorporated into the predicted risk in this risk calculator. The outcome “risk of any PCa” was used; “risk of high-grade PCa” was left out of consideration.

### 2.5. MRI

A bi-parametric MRI protocol was scanned on a 3 Tesla scanner using a “pelvic-phased array coil” (Magnetom Skyra, Siemens Nederland B.V., The Hague, The Netherlands). The sequences included sagittal, coronal, and axial T2-weighted images, along with axial diffusion-weighted images. Images were scored according to the Prostate Imaging Reporting and Data System (PIRADS) version 2 [15]. Since Dynamic Contrast Enhanced (DCE) series were not applied, it was not possible to upgrade peripheral zone PIRADS 3 lesions in case of an abnormal DCE. Scans were reviewed by experienced uro-radiologists (>1000 scans) and generally the principle of double-reading was applied in the multidisciplinary meeting, in which all patients with PIRADS ≥ 3 were discussed for biopsy indication and strategy.

### 2.6. Analysis

With the reference pathway as the golden standard, the outcomes of a hypothetical full MRI-based pathway were studied, in which:

1. Indication for biopsy was based solely on MRI findings, omitting systematic biopsies in patients with normal MRI but abnormal risk-calculator scores.
2. Biopsy in abnormal MRI was only performed using targeted biopsies, omitting additional systematic cores.

The following outcomes were compared qualitatively between the reference pathway and the hypothetical fully MRI-based pathway: number of biopsy indications, number of biopsy cores, detection rate of significant tumors, and detection rate of insignificant cancers.

In men with missing biopsy outcomes (when not compliant with the indication for prostate biopsy), missing results were entered/extrapolated using cancer detection rates of men who did undergo biopsy as per protocol.

## 3. Results

**Figure 1:**
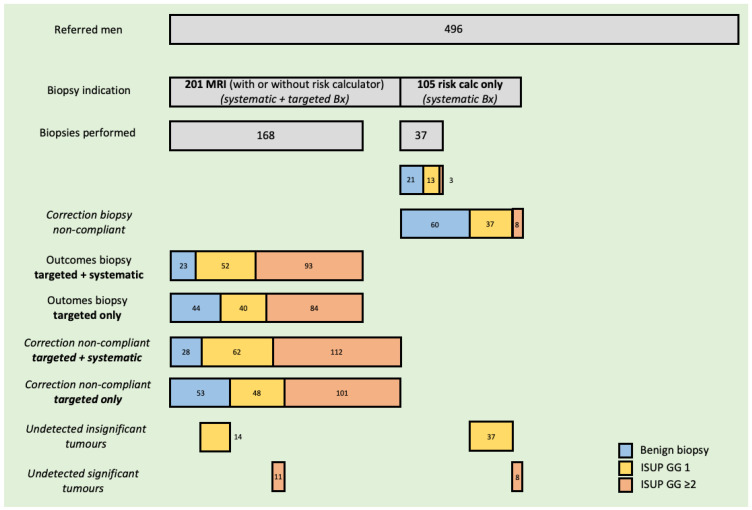
Extrapolated to 100% biopsy indication compliance, 120 (8 + 112) significant and 99 (37 + 62) insignificant tumors would have been detected. Omission of systematic biopsies in cases with normal MRI (PIRADS 1–2) but abnormal risk-calculator (≥20%) would miss 7% (8/120) of significant and 37% (37/99) of insignificant tumors. Targeted biopsy only and omission of systematic biopsies in cases with abnormal MRI (PIRADS 3-4-5) would miss 9% (11/120) of significant and 14% (14/99) of insignificant tumors.

A total of 496 patients were included in the cohort. Figure 1 presents the main study design and outcomes. Table 1 presents patient characteristics of the reference cohort. Of the total, 306 patients (62%) had an indication for biopsy: 141 (28%) based on both the risk-calculator score ≥ 20% and MRI PIRADS ≥ 3, 60 (12%) based on PIRADS ≥ 3 only, and 105 (22%) based on risk-calculator only. See Figure 1.

Of the 306 patients with an indication for biopsy, 205 (67%) underwent biopsies. Biopsy indication compliance was 87% (125/141) in men with a risk-calculator score ≥ 20% and abnormal MRI, 72% (43/60) in patients with MRI PIRADS 3-4-5 ONLY, and 35% in patients with ONLY risk-calculator score ≥ 20%.

Significant PCa was found in 96 patients (extrapolated to a perfect biopsy indication compliance, this would translate to 120 patients (24%)). Insignificant PCa was found in 65 patients (extrapolated to a perfect biopsy indication compliance, this would translate to 99 patients (20%)).

### 3.1. Omission of Systematic Biopsies in Normal MRI but Risk-Calculator ≥ 20%

As depicted in Figure 1, when the indication for prostate biopsy would have been solely made on an abnormal MRI, without considering an abnormal risk-calculator, the biopsy indication would have been dropped in 34% (105/306) of referred men. Within our cohort, three significant cancers would have been left undetected in this group, all GG 2. These patients had prostate risk-calculator scores of 26%, 40%, and 43%, and one patient had an abnormal DRE. 

Extrapolated to a perfect biopsy-indication compliance, 8 significant tumors would have been left undetected, 7% of (8/120) the total. Of the total number of GG 1 cancers, 37% (37/99) would not have been diagnosed.

### 3.2. The Omission of Systematic Biopsies in Case of Abnormal MRI Findings

When patients with an abnormal MRI only would have received targeted biopsies, instead of combined targeted plus systematic biopsies, the number of biopsy cores per patient would have been reduced by >75% (2–3 per patient versus a median of 12).

See Figure 1. Within our cohort, this strategy would have resulted in missing significant PCa in 9 (9%) patients, of whom 6 patients had GG 2 and 3 patients had GG 4. Table 2 presents the patient characteristics of these 9 patients. Two patients could be considered biopsy errors as they had an abnormal DRE, PSA > 10, and PIRADS 5. In 6 out of 9 (67%) patients in whom the systematic biopsies yielded additional value to the targeted biopsies, this was on the ipsilateral side of the MRI index lesion.

Extrapolated to a perfect biopsy indication compliance (total N = 120), 11 (9%) significant tumors would have been left undetected. Of the total number of GG 1 cancers, 14% (14/99) would not have been diagnosed.

## 4. Discussion

This study presents the outcomes of a hypothetical diagnostic pathway for patients referred for a suspicion of PCa who undergo direct bpMRI and risk-calculator, in whom systematic biopsy cores would have been omitted. This corresponds to a scenario in which the only biopsy indication is a PI-RADS ≥ 3 lesion, which is followed by a targeted biopsy-only strategy. This approach was compared to the reference pathway utilized at our hospital, in which an aberrant risk-calculator score also served as an indication for biopsy, and when possible, a combination of targeted and systematic biopsy cores was used. By omitting systematic biopsies in men with a prostate risk calculation of ≥20% but normal MRI, the indication for biopsy would have been dropped by 34%, with 7% of the total significant tumors and 37% of the insignificant tumors remaining undetected. By only taking targeted biopsies and omitting the systematic biopsies in men with an MRI PIRADS ≥3, 75% fewer biopsy cores would have been taken, with 9% of the total significant tumors and 14% of insignificant tumors remaining undetected. Most of the tumors left undetected were GG 2 (Gleason 3 + 4 = 7) cancers on the ipsilateral side of the MRI index lesion. 

The findings in this large cohort are somewhat more favorable than those published in the recent literature. Sathianathen et al. reported a combined negative predictive value of MRI for significant PCa (when defined as GG ≥ 2) of 90.8% [3]. The interpretation of this is that around one in 10 men with a normal MRI still harbors significant PCa. In the current cohort, this percentage was only one in 14. It is important to note that the MRI does not detect all prostate tumors. Anterior, small, ISUP GG 1–2 lesions, and “sparse” prostate tumors, which display ADC and T2 values similar to tumors in the normal peripheral zone tissue, might remain undetected [16,17]. Ploussard et al. found that combined systematic plus targeted biopsies detect around 10% more significant PCa when compared to targeted biopsies only [8]. This percentage of 10% was confirmed in our cohort. The 4M study also provides a valid comparison, because this study analyzed the impact of a similar MRI-guided pathway for biopsy indication and targeted biopsies in a Dutch prospective study [11]. Table 3 presents a comparison between the current study cohort and the 4M study. This comparison is mainly “hypothesis generating”. Numerous potential differences could account for the observed dissimilarities, including: diagnostic accuracy of MRI, quality and method of targeted and systematic biopsies, patient selection and PCa prevalence, study design, etc. 

Not detecting significant cancers is undesirable, but should be nuanced. First, of the significant PCas left undetected by targeted biopsy cores only, some appear truly “missed” as these had a combination of very unfavorable characteristics. Also, in 6 out of 9 significant PCas left undetected by targeted cores only, significant cancer in the systematic cores was found on the same side as the MRI index lesion. Further experience with the (cognitive) targeting procedure, or the use of peri-lesional biopsy cores could partly correct for this. Second, out of the three GG 4 tumors (all Gleason 4 + 4 = 8) that were left undetected by targeted biopsy cores alone, the targeted cores detected GG 1 cores in one of these cases. Out of the six GG 2 tumors that were left undetected by the targeted cores, the targeted cores detected GG 1 in five of these cases. These patients would not have been discharged from follow-up, but would have been included in the strict follow-up of an active surveillance protocol. Third, men with an elevated PSA but normal MRI are generally followed up through regular PSA checks. PSA changes would most likely detect any remaining significant tumors before the window of curability would have been missed. For men with an abnormal MRI but negative biopsies (in this cohort this comprised 3 out of 9 cancers left undetected, translating to 2–3% of the total detected significant cancers), regular checks will also be started, or additional imaging such as PSMA-PET/CT may be applied. The potentially unfavorable impact of a delayed diagnosis and consequent treatment is likely to be limited [18]. Also, intermediate-risk tumors not visualized on MRI have a more favorable prognosis when compared to visible lesions [19].

The strength of this analysis is the size and the fact that direct bpMRI was performed in all unselected referred men. Different limitations should be highlighted. First, the pathway used as a reference also provides relative outcomes only. Template biopsies, prostatectomy specimens, or clinical follow-up outcomes would impact PCa findings. Second, biopsy-indication compliance was imperfect, making it necessary to artificially extrapolate the absolute cancer detection rates if 100% of patients with a biopsy indication would indeed have received biopsies. For this correction, outcomes of patients from the same biopsy-indication group were used. This introduces a bias, but as mainly patients with a higher risk complied with the biopsy indication, and is likely to result in a less favorable number of cases left undetected. Third, the cohort only includes patients referred by the GP. Current results may not be applicable to patients entering the diagnostic pathway via another route such as screening. Fourth, biopsies were targeted according to cognitive MRI-TRUS fusion, using a freehand technique. The future-trial showed comparable outcomes for software-fusion or in-bore techniques, although this trial included men with previous negative systematic biopsies only [12]. Fifth, a biparametric MRI protocol was used, not the multiparametric as originally recommended by the PIRADS steering committee. However, different studies and meta-analyses have been published recently, indicating comparable sensitivity and specificity for the detection of significant PCa [20,21]. Also, it is worth noting that the DCE’s efficacy in detecting clinically significant PCa is particularly pronounced with 1.5 Tesla scanners, rather than the 3 Tesla scanners utilized in this study [22]. Furthermore, employing experienced uro-radiologists in this study should ensure no discrepancy in evaluating index lesions on bpMRI compared to mpMRI [23]. Moreover, the PIRADS steering committee has officially stated that bpMRI is an acceptable option in biopsy-naïve patients [24]. A quality scoring system for bpMRI, similar to the PI-QUAL developed specifically for mpMRI, could further optimize this pathway and assist in determining the reliability of the MRI quality when deciding on initial biopsy [25]. Sixth, a strategy using targeted cores only will also impact further management decisions. This includes risk-indication for active surveillance versus radical therapy, active surveillance follow-up biopsies, risk stratification for dissemination imaging, nerve-sparing planning during prostatectomy, indication or lymph node dissection, etc. Finally, larger patient numbers would further validate the current results, although these are generally in line with the published literature.

A diagnostic pathway using imaging as the main indication for biopsy using targeted cores only is a rational strategy; positive MRI is highly predictive of significant PCa and biopsy cores are more efficient due to targeting, especially when addressing tumors in the anterior part of the gland [26]. Contrarily, men with a negative MRI and other indications for biopsy have an a priori lower risk of significant PCa, and biopsy cores can only be applied systematically. Performing more cores will per definition increase cancer detection rates, but it can be questioned whether this “advantage” is in balance with the higher costs and burden (more insignificant PCa, patient burden, time, costs, infections, etc.) [27]. Furthermore, standard follow-up or active surveillance may cover the limited number of cases of significant PCa that were left undetected.

**Figure 2:**
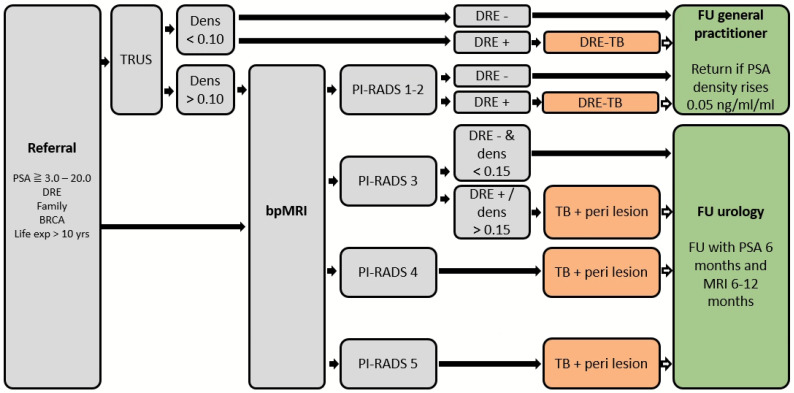
Example of a risk-based pathway for suspected prostate cancer, MRI-guided and using targeted biopsies only. BRCA—breast cancer (gene mutation); Bp—biparametric; Dens—density; DRE—digital rectal examination; Exp—expectancy; FU—follow-up; MRI—magnetic resonance-imaging; PIRADS—prostate imaging reporting and data system; PSA—prostate-specific antigen; PSMA—prostate-specific membrane antigen; TB—targeted biopsy; TRUS—transrectal ultrasound.

Different aspects of the diagnostic pathway may be further optimized. First, initially omitting biopsies in PIRADS 3 lesions, would avoid 11% of the indications for biopsy, miss 11% of the GG 1 tumors, and miss 3% of the significant PCas. If biopsies would only be omitted in PIRADS 3 lesions with PSA density < 0.15 ng/mL/mL AND normal DRE, these percentages would be 6%, 3%, and 0%, respectively (no significant PCa left undetected). Alternatively, following the approach proposed by Boschheidgen et al., performing a follow-up MRI after 12–24 months for patients with PIRADS 3 could safely omit the need for initial biopsies without missing significant PCa: all patients with significant PCa demonstrated an increase in both PIRADS and PSA density during follow-up. In contrast, patients without PCa showed a significant decrease in PIRADS during follow-up [28]. Second, of the 73 men with an abnormal DRE, 13 (18%) had MRI PIRADS 1–2. Corrected for biopsy compliance, 3 patients with GG 1 and 3 patients with significant PCa would be found in this group. Performing biopsies in this small group of patients with negative MRI but abnormal DRE would lead to 4% more biopsies, 3% more GG 1, and 3% significant PCas. Third, pre-selection for MRI could be applied. All patients in our reference pathway received direct MRI. Often, risk calculators and MRIs are sequenced, i.e., an MRI is only performed after the risk calculation has been found abnormal. Although this is a sensible strategy, it should be remembered that this is a different approach as applied in well-known MRI studies such as PROMIS and PRECISION in which a PSA threshold is applied [2,7]. Reesink et al. previously showed that sequencing biopsy risk stratification tools indeed avoid MRI scans, but that more significant PCas are being left undetected (risk-calculator threshold 20%: 53% fewer MRIs, but 19% significant PCas left undetected; risk-calculator threshold 12.5%: 31% fewer MRIs, but 7% significant PCas left undetected) [5]. Another approach is the incorporation of MRI data into risk calculators. However, these risk calculators have shown to be effective especially in men who have been previously biopsied. Additionally, these calculators were evaluated using the first version of the PIRADS, with lower sensitivity than the version now recommended to use by the EAU guidelines [1,29,30]. Any pathway applying MRI first outperforms the diagnostic accuracy of a pathway in which a risk calculator is used as the first step. Finally, per-lesional biopsy cores may provide an acceptable middle way between bilateral systematic biopsies and targeted biopsy only [31]. Figure 2 presents a possible MRI-guided pathway incorporating the above suggestions.

The diagnostic pathway for the suspicion of PCa is currently a chain of clinical steps in which GP, urologist, radiologist, nuclear physician, and pathologist work closely together. The introduction of MRI and targeted biopsies has improved the pathway but also has introduced diagnostic steps highly dependent on the expertise and quality of those involved. The quality of every step in the process has a downstream impact. Within the confinements of guidelines recommendations, every center can make specific choices in the diagnostic pathway. However, it is vital to have insight into the center-specific performance of MRI and biopsy outcomes, in order to compare and improve. Comparison of centre-specific data with national results, allows improvement of the quality of the diagnostic pathway and the perfect pathway can be further optimized.

## 5. Conclusions

In men referred for suspicion of PCa, direct bpMRI and targeted biopsy allows for refinement of biopsy indication and strategy. Omission of the indication for systematic biopsies in cases with a normal MRI would have resulted in a decrease in biopsy indication of 34%, but 7% of the significant tumors would have been left undetected, as well as 37% of the insignificant tumors. Furthermore, the omission of systematic biopsies in cases with an abnormal MRI and taking only targeted biopsies would have resulted in a decrease in the number of cores of 75% per procedure, but 9% of the significant tumors would have been missed, as well as 14% of the insignificant tumors. These findings may be used to balance and optimize the diagnostic pathway for PCa. Adequate insight into patient characteristics, MRI, and biopsy performance, combined with prospective data registration are required for a center before the omission of systematic biopsies is considered. Future prospective studies are needed to determine the safety and outcomes of these approaches.

## Figures and Tables

**Table 1:** Patient characteristics of reference cohort.

	Total	
Characteristics	Median	(IQR)
	496	
**Age (yr)**	68	(62–73)
**PSA (ng/mL)**	6.5	(5.1–9.3)
**Prostate volume (mL)**	50	(35–70)
	**n**	**(%)**
**DRE**		
Normal	342	(69)
Abnormal	85	(17)
Unknown	69	(14)
**Risk-calculator categories**		
<12.5%	153	(31)
12.5–20%	110	(22)
>20%	233	(47)
**PIRADS**		
1	32	(7)
2	263	(53)
3	33	(7)
4	78	(16)
5	90	(18)

DRE—digital rectal examination; IQR—interquartile range; PIRADS—prostate imaging reporting and data system; PSA—prostate-specific antigen.

**Table 2:** Patient characteristics of nine patients in whom targeted biopsies found no cancer or ISUP grade group 1 (Gleason 3 + 3 = 6), while systematic biopsies showed ISUP grade group ≥2 (Gleason 3 + 4 = 7).

Age	PSA	Vol	DRE	Risk Calc	#TB	TBpos	TBGG	#SB	SBpos	SBGG
71	10.1	30	cT2	71	2	2	1	8	7	2
72	10.6	55	cT1c	28	3	3	1	10	5	2
60	11.1	56	cT1c	29	2	0	0	10	3	4
67	4.5	46	cT1c	12	2	0	0	8	7	2
68	4.2	55	cT2	16	3	2	1	7	5	2
56	5.6	32	cT1c	27	3	3	1	10	5	2
65	5.2	50	NR	13	3	0	0	8	2	4
69	29.7	48	cT2	86	2	2	1	8	4	2
60	5	12	cT1c	65	3	2	1	9	3	4

DRE—digital rectal examination; NR—not reported; Risk ind—risk-calculator percentage of prostate cancer; SB—systematic biopsy; SBGG—systematic biopsy cores maximal ISUP grade group; SBpos—positive systematic biopsy cores; TB—targeted biopsy cores; TBGG—targeted biopsy cores maximal ISUP grade group; TBpos—positive targeted biopsy cores; Vol—prostate volume.

**Table 3:** Comparison of the present study cohort with the 4M study (10).

	Cohort	4M Study
Patients		
N	496	626
Mean age	68 yr	65 yr
Mean PSA	6.5 ng/mL	6.4
Mean PSA density	0.13 ng/mL/mL	0.11 ng/mL/mL
Abnormal DRE	20%	28%
**Biopsies**		
Biopsy indication	Risk-calculator ≥20% and/or MRI PIRADS 3-4-5	PSA ≥3.0 and/or MRI PIRADS 3-4-5
Biopsy strategy—targeted	Cognitive “freehand”, fusion 2–3 cores per lesion	“In-bore”, 2–4 cores per lesion
Biopsy strategy—systematic	8–10–12 per volume	12 cores
Biopsy indication	62%	100%
Significant PCa	24%	30%
Insignificant PCa	18%	23%
**MRI**		
Protocol	T2, DWI	T2, DWI, DCE
PIRADS 1–2	59%	49%
PIRADS 3	7%	6%
PIRADS 4	16%	22%
PIRADS 5	18%	23%
**Significant PCa missed by omission systematic cores (of total significant PCa)**		
In normal MRI cases	7% (8/120 *)	5% (10/200)
In abnormal MRI cases (targeted-only)	9% (11/120 *)	11% (21/180)

* Extrapolated for biopsy indication non-compliance. DCE—Dynamic Contrast-Enhanced imaging; DRE—digital rectal examination; DWI—Diffusion Weighted Imaging; MRI—magnetic resonance imaging; PCa—prostate cancer; PIRADS—prostate imaging reporting and data system; PSA—prostate-specific antigen; T2—T2-weighted image.

## Data Availability

The data presented in this study are available on request from the corresponding author. The data are not publicly available due to privacy and ethical reasons.
